# Threat history controls flexible escape behavior in mice

**DOI:** 10.1016/j.cub.2022.05.022

**Published:** 2022-07-11

**Authors:** Stephen C. Lenzi, Lee Cossell, Benjamin Grainger, Sarah F. Olesen, Tiago Branco, Troy W. Margrie

**Affiliations:** 1 Sainsbury Wellcome Centre for Neural Circuits and Behaviour, University College London, 25 Howland Street, London W1T 4JG, UK

**Keywords:** suppression of escape, looming stimulus, mouse, flexible, adaptation, decision-making

## Abstract

In many instances, external sensory-evoked neuronal activity is used by the brain to select the most appropriate behavioral response. Predator-avoidance behaviors such as freezing and escape^1^^,^^2^ are of particular interest since these stimulus-evoked responses are behavioral manifestations of a decision-making process that is fundamental to survival.^3^^,^^4^ Over the lifespan of an individual, however, the threat value of agents in the environment is believed to undergo constant revision,^5^ and in some cases, repeated avoidance of certain stimuli may no longer be an optimal behavioral strategy.^6^ To begin to study this type of adaptive control of decision-making, we devised an experimental paradigm to probe the properties of threat escape in the laboratory mouse *Mus musculus*. First, we found that while robust escape to visual looming stimuli can be observed after 2 days of social isolation, mice can also rapidly learn that such stimuli are non-threatening. This learned suppression of escape (LSE) is extremely robust and can persist for weeks and is not a generalized adaptation, since flight responses to novel live prey and auditory threat stimuli in the same environmental context were maintained. We also show that LSE cannot be explained by trial number or a simple form of stimulus desensitization since it is dependent on threat-escape history. We propose that the action selection process mediating escape behavior is constantly updated by recent threat history and that LSE can be used as a robust model system to understand the neurophysiological mechanisms underlying experience-dependent decision-making.

## Highlights

• Individually housed, but not group-housed, mice show robust escape to looming stimuli
• Mice can learn to suppress escape, and LSE memory is long lasting
• LSE is not a general adaptation since it is stimulus specific
• LSE is not simply habituation and is dependent on recent threat-escape experience

## Teaser

Lenzi et al. develop a paradigm in mice that induces suppression of otherwise robust escape to looming stimuli. This learned suppression of escape (LSE) is long lasting, stimulus specific, and under cognitive control since it is dependent on threat-escape memory. LSE is a unique model system to study the neuronal basis of flexible decision-making.

## Results

An ethological approach that links sensory stimuli in the environment to behavior is necessary for understanding how real-world experience shapes behavior selection.^7^ Predator avoidance behaviors such as escape are particularly relevant since they are evolutionarily important^5^^,^^8^ and well characterized at the neural level.^3^^,^^9^, ^10^, ^11^, ^12^, ^13^, ^14^, ^15^ When faced with threats, escape or freezing responses can be rapidly selected based on recently acquired information about the surrounding environment such as distance to shelter.^4^ However, over longer timescales, an innately threatening stimulus may be learned to be non-threatening, and in such circumstances, the ongoing selection of escape behavior would be disadvantageous. To determine how threat and environmental experience influences decision-making over such timescales, we first sought to establish a high-throughput, robust assay of escape behavior that could form the backbone of a paradigm for understanding the adaptive control of this kind of action selection.

### Robust escape behavior in mice individually housed for at least 2 days

**Figure 1:**
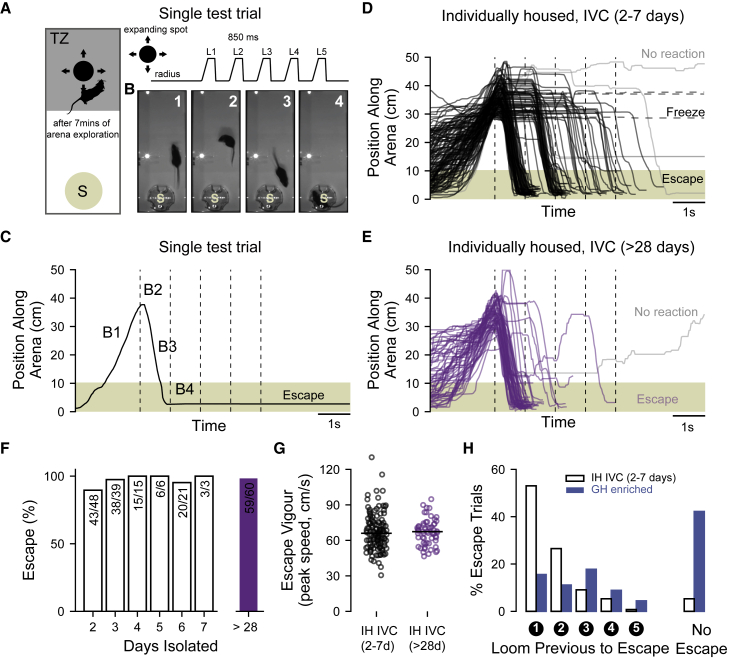
Robust escape behavior in individually housed mice (A) Schematic showing the experimental arena containing a circular-shaped shelter and the threat zone (TZ), above which looming stimuli were presented. Looming stimuli were composed of 5 sequential expanding spots. (B) Image frames from a single trial in chronological order showing the mouse entering the threat zone (B1), turning towards the shelter after stimulus onset (B2), fleeing (B3), and finding refuge inside the shelter (B4). (C) A single example trace of the position of the mouse from the trial shown in (B). (D) Population data showing positional traces of all trials from all mice housed in IVCs for between 2–7 days (traces are classified as escape [solid line], freezing [dashed line], or no reaction [solid grey line]). (E) Population data showing positional traces of all trials from all mice housed in IVCs for at least 28 days. (F) Overall percentage of trials classified as escape according to IVC housing period. (G) Peak speed of escape plotted for all trials where escape responses were observed in mice singly housed for either 2–7 days (black, n = 44 mice) or more than 28 days (purple, n = 20 mice). Horizontal lines indicate the median values for each group. (H) Left: percentage of escape trials plotted against the number of looming spots presented prior to escape for all mice isolated for between 2–7 days versus all mice group housed in the enriched pen. Only trials that were classified as escapes are shown. Right: percentage of all trials that were classified as no escape and includes freezing trials.

Commercially bred C57BL/6J mice were raised in small litters until weaning (3 to 4 weeks of age) and then were individually housed (IH) in a standard size unenriched individually ventilated cage (IVC) (IH IVC group). Individual mice were housed for a minimum of 2 days and up to 1 month prior to testing and then transferred to a behavioral arena (Figure 1A) where we could digitally track the location of individuals before, during, and after overhead presentation of an expanding spot presented on a computer monitor positioned directly above (Figures 1A–1C). For mice housed in IVCs for between 2 and 7 days, we observed extremely high levels of escape probability (2 days individually housed, n = 16 mice, 43/48 [89.5%] trials; 3 days, n = 13, 38/39 [97.4%] trials; 4 days, n = 5 mice, 15/15 [100%] trials; 5 days, n = 2 mice, 6/6 [100%] trials; 6 days, n = 7 mice, 20/21 [95.2%] trials; 7 days, n = 1 mice, 3/3 [100%] trials; p > 0.05 between all groups, Fisher’s exact test). The average probability and peak speed of escape of mice individually housed for between 2 and 7 days was indistinguishable from those mice housed in IVCs for at least 28 days (Figures 1D–1G; probability: IH IVC 2–7 days, n = 44 mice, 125/132 [94.7%] trials versus IH IVC 1 month, n = 20 mice, 59/60 [98.3%] trials, p = 0.44 versus IH enriched, n = 6 mice, 17/18 [94.4%] trials, p = 1.00, Fisher’s exact; median peak speed: IH IVC 2–7 days = 66.0 cm/s [interquartile range (IQR) = 18.2 cm/s, n = 125 escapes] versus IH IVC 1 month = 67.4 cm/s [IQR = 14.4 cm/s, n = 59], p = 0.64 versus IH enriched = 72.7 cm/s [IQR = 20.0 cm/s, n = 17], p = 0.12, Wilcoxon’s rank-sum test).

In contrast, weaned mice that continued to be group housed (GH) (n = 20 per group) for at least 28 days in a large, enriched pen (Figure S1A) were found to have highly variable trial-by-trial responses compared to mice maintained alone for the same period in the same enriched pen (Figures 1H, S1B, and S1C). On average, group-pen-housed mice exhibited a significantly lower overall escape probability compared to individually housed mice raised in either the same enriched pen (Figure S1C; GH enriched, n = 15 mice, 26/45 [57.8%] trials versus IH enriched, n = 6 mice, 17/18 [94.4%] trials, p = 0.006, Fisher’s exact test) or an IVC (Figure 1H; GH enriched, n = 15 mice, 26/45 [57.8%] trials versus IH IVC, n = 20 mice, 59/60 [98.3%] trials, p < 0.0001, Fisher’s exact test). Also, the observed overall high escape probabilities (>90%) of mice individually housed in the enriched pen or IVCs were not different from each other (IH enriched, n = 6 mice, 17/18 [94.4%] trials versus IH IVC, n = 20 mice, 59/60 [98.3%] trials, p = 0.41, Fisher’s exact). The number of test trials on which group-housed mice escaped varied from zero to three with the number of mice approximately evenly distributed among those possibilities (Figure S1D; p = 0.51; chi-squared test) while all individually housed mice exhibited flight responses on either two or three of the three test trials (Figure S1D; IH enriched: p = 0.01; IH IVC: p < 0.0001; chi-squared tests). We also found a significant reduction in both the median latency to escape (GH enriched = 1.98 s [IQR = 1.73 s, n = 26 escapes] versus IH enriched = 0.24 s [IQR = 0.39 s, n = 17 escapes], p = 0.002 versus IH IVC = 0.16 s [IQR = 0.15 s, n = 59 escapes], p = 3.8x10^−9^, Wilcoxon’s rank-sum test) and peak speed of escape (Figure S1E; mean peak speed; GH enriched = 47.9 cm/s [IQR = 15.4 cm/s, n = 26 escapes] versus IH enriched = 72.7 cm/s [IQR = 20.0 cm/s, n = 17 escapes], p = 2.7×10^−6^ versus IH IVC = 67.4 cm/s [IQR = 14.4 cm/s, n = 59 escapes], p = 1.2×10^−8^, Wilcoxon’s rank-sum test) for group-housed compared to individually housed animals. Based on escape probability, latency, and speed (GH enriched, robustness metric = 13.7 a.u. [IQR = 25 a.u., n = 15 mice] versus IH enriched = 203 a.u. [IQR = 175 a.u., n = 6], p = 0.004 versus IH IVC = 38 1a.u. [IQR = 335 a.u., n = 20], p = 6.8×10^−7^, Wilcoxon’s rank-sum test), social isolation for as little as 2 days is therefore sufficient to ensure a high baseline level of escape behavior.

### Suppression of escape in loom-naive, individually housed mice

Repeated exposure to potential predatory stimuli that are subsequently learnt to be non-threatening causes a reduction to the flight initiation distance^16^^,^^17^ and even behavioral reversal from avoidance or retreat to predation.^6^ Such adaptive behavior, for example, underlies the domestication of wild animals and represents a form of cognitive control and learning^18^ that can maximize access to resources and conspecifics and enhance the evolutionary fitness of the individual. Based on our behavioral data above, the escape circuitry in mice appears amenable to adaptive control by factors in the environment, but can the history of threat experience itself influence future escape decisions?

**Figure 2:**
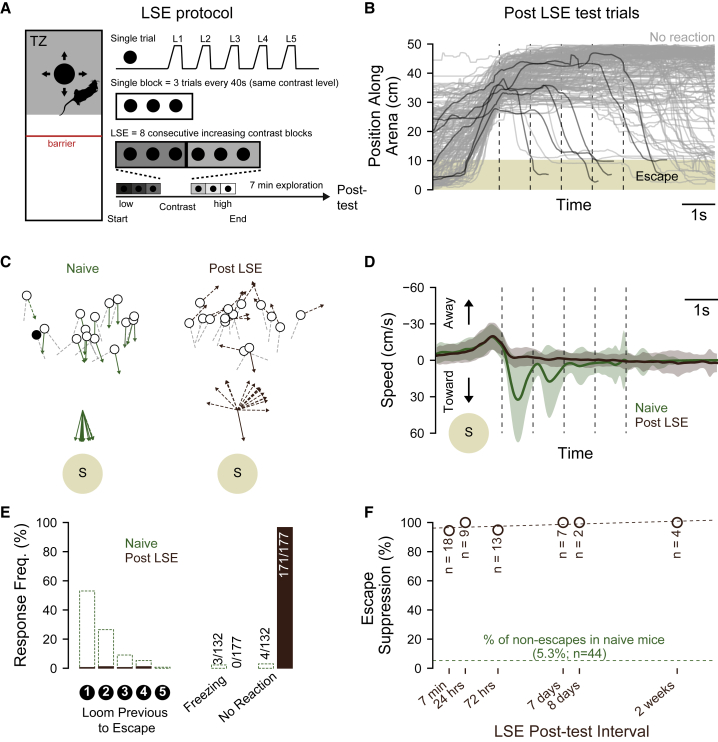
Rapid and prolonged escape suppression in loom-naive mice (A) Schematic of the stimulus protocol used to elicit learned suppression of escape (LSE). (B) Population data showing positional traces for test trials performed after the LSE protocol. (C) Top: average heading direction (20 frames prior to stimulus onset, grey dashed line) and mouse position (circles) at the onset of stimulus presentation for each mouse and each trial. Minimum heading angle relative to shelter recorded following stimulus onset (STAR Methods) is represented by the “arrow.” Solid lines with an arrowhead indicate escape trials. Filled circles indicate a freezing response. Bottom: post-stimulus trajectories (from above) for all mice and all trials plotted relative to shelter location. (D) Population average velocity traces (solid lines) and standard deviation (shaded area) for naive and LSE mice. (E) The percentage of trials classified as escape, grouped by most recent looming spot preceding escape initiation (left) and the percentage of responses classified as freezing (middle) and no reaction (right) for each experimental group. (F) The probability of escape suppression in mice at various time points following the LSE protocol (brown circles) with a line of best fit (dashed brown line). Each mouse (n = 53) was tested at a single time point only. The dashed green line indicates non-escape probability of naive control mice.

To assess this, we developed a paradigm whereby mice that had been individually housed in IVCs for at least 2 days were introduced directly to a threat zone that was partitioned from the remaining arena. Mice were then presented a set of 24 looming stimuli (one every 40 s) with a gradually increasing contrast while keeping the luminance of the looming black spot constant (Figure 2A). After the protocol, and in contrast to naive animals, we observed that the vast majority of learned suppression of escape (LSE)-treated mice did not escape to the test stimulus (Figure 2B; n = 59 mice, 171/177 [96.7%] trials). Qualitatively, the presence of a shelter did not appear to impact general exploratory behavior or coverage of the threat zone during the protocol. The presence of a shelter also had no significant effect on the induction of LSE (Figures S2A–S2C; fraction of post-LSE escapes with shelter absent, n = 58 mice, 6/177 [3.4%] trials versus with shelter present n = 4 mice, 0/12 [0%] trials, p = 1.00).

Compared to naive animals, LSE mice showed no reorientation of their direction heading toward shelter following the onset or during presentation of the test stimulus (Figure 2C; median absolute change in heading direction: LSE = 47.8° [IQR = 23.9°, n = 5 mice, n = 15 trials] versus naive = 153.3° [IQR = 55.5°, n = 5 mice, 15 trials], p < 0.001, Kuiper’s test). Likewise, despite having very similar baseline locomotion speeds, only naive animals showed an increase in locomotion speed towards shelter (Figure 2D; median absolute peak speed 1s before loom: LSE = 26.2 cm/s [IQR = 13.9 cm/s, n = 59 mice, n = 177 trials] versus naive = 25.2 cm/s [IQR = 15.1 cm/s, n = 44 mice, n = 132 trials], p = 0.25; median absolute peak speed in 1 s after loom: LSE = 17.1 cm/s [IQR = 11.3 cm/s, n = 59 mice, n = 177 trials] versus naive = 50.9 cm/s [IQR = 59.1 cm/s, n = 44 mice, n = 132 trials], p < 0.0001, Wilcoxon’s rank-sum test). In LSE-treated mice, the absence of escape (Figures 2B and 2E; LSE: n = 59 mice, 171/177 [96.6%] trials versus naive: n = 44 mice, 7/132 [5.3%] trials, p < 0.0001, Fisher’s exact test) or alternative defensive behaviors, such as freezing (LSE: 0/177 trials versus naive: 3/132 trials, p = 0.07, Fisher’s exact test), meant that there was no observable stimulus-evoked response on 97% of trials. The LSE protocol therefore rapidly induces a robust suppression of loom-evoked escape responses to the test stimulus.

### LSE is long lasting

If LSE is important for optimizing the success of other ethologically important tasks such as predation,^6^ then one might expect it to be expressed long term. To explore this, we varied the interval between the LSE protocol and the test trials from between 7 min to 2 weeks. LSE was found to induce robust suppression at all time points tested (Figure 2F; post-LSE test time point: 7 min, n = 18 mice, 51/54 [94.4%] trials; 24 h, n = 9 mice, 27/27 [100%] trials; 72 h, n = 13 mice, 37/39 [94.9%] trials; 7 days, n = 7 mice, 21/21 [100%] trials; 8 days, n = 2 mice, 6/6 [100%] trials; 2 weeks, 4 mice, 12/12 [100%] trials; all p < 0.0001 versus naive = 7/132, Fisher’s exact test) indicating that LSE plasticity is not only rapidly induced but persistent enough to have long-lasting influence on behavior selection.

### LSE is stimulus specific and depends on threat experience

In some scenarios, it has been observed that reduced responsiveness to artificial threatening stimuli can induce a general habituation at the location at which those stimuli were experienced.^19^^,^^20^ This raises the possibility that LSE could reflect learning about particular safe locations or contexts rather than specific types of threat stimuli. We therefore tested whether mice that suppress escape to looming stimuli exhibit reduced predator avoidance responses to other threatening stimuli in the same area of the arena.

**Figure 3:**
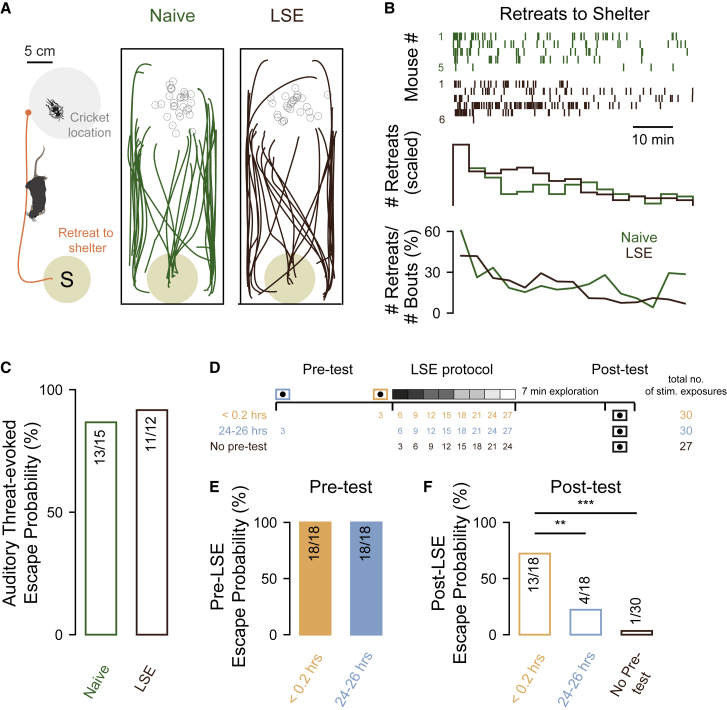
LSE is stimulus specific and dependent on recent threat history (A) Left: schematic of the experimental paradigm in which a mouse was free to interact with a novel cricket tethered to the center of the threat zone (grey circle indicates the upper limit of the range of cricket at retreat onset). An example trace of a retreat to shelter is represented by the orange line, starting from the moment the mouse enters the regional limit of the tether point (orange circle). Right: positional traces for all mice and all bouts exhibiting returns into or near to shelter for each experimental group. Small grey circles indicated the position of the cricket at the onset of retreat for each bout. (B) Top: raster plots of the onset times of all retreats for naive (green, n = 5) and LSE (brown, n = 6) mice. Middle: histograms of retreat onset times scaled to the first 4 min time bin. Bottom: line plots of the fraction of all bouts that result in a retreat, over time. (C) Bar plot of escape probability to auditory stimuli in loom-naive (green) and LSE (brown) mice. (D) Schematic showing the experimental paradigm for assessing the effect of prior exposure to a pre-test at either <0.2 h (orange) or 24–26 h (blue) before LSE and the subsequent total number of stimulus exposures (right). (E) Pre-test escape probability of mice tested <0.2 h before or 24–26 h before LSE. (F) Post-LSE escape probability for the <0.2 h and 24–26 h, and no pre-test experimental groups.∗∗, ∗∗∗ indicate p values of less than 0.01 and 0.001, respectively.

When mice are exposed to novel live prey for the first time, in many instances, they can exhibit avoidance behavior,^6^ including retreats to shelter (Figure 3A). We therefore tethered live crickets to the center of the threat zone and compared the avoidance behavior of naive and LSE mice. LSE mice exhibited high levels of avoidance behavior to the cricket (Figure 3B), and both the number of initial interactions (median number of bouts in first 10 min for naive mice = 14 [IQR = 13, n = 5 mice] versus median for LSE mice = 21.5 [IQR = 5.3, n = 6 mice], p = 0.65, Wilcoxon rank-sum test) and retreats to shelter (median number of retreats to shelter in first 10 min for naive mice = 10 [IQR = 5] versus for LSE = 8.5 [IQR = 7.75], p = 0.93) were not different between the two groups (Figure 3B). Similarly, a different artificial threat stimulus, such as auditory pink noise presented over the threat zone, was equally effective in producing escape in LSE-treated and naive mice (Figure 3C; naive, n = 5 mice, 13/15 [86.7%] trials versus LSE, n = 4 mice, 11/12 [91.7%] trials, p = 1.00, Fisher’s exact test). These data show that suppression of threat avoidance is based on a stimulus-specific experience rather than a location-related one.

So what factors contribute to the suppression of escape? In a final set of experiments, we explored whether LSE itself was simply dependent on repeated exposure to the looming stimulus. On the one hand, simple forms of non-associative learning such as habituation have been observed in some species following repeated presentation of visual stimuli known to elicit escape.^21^ On the other hand, studies in the wild indicate that prey animals can alter their threat assessment of environments based on the outcome of past encounters,^18^ suggesting that escape is under cognitive control and potentially influenced by threat escape history. To test this idea, we devised an experiment in which three groups of mice (that had all been individually housed in IVCs for at least 2 days) were exposed to a similar number of looming stimuli with slightly different temporal histories (Figure 3D). For two of the groups, mice were presented with three standard-test looming stimuli either 24–26 h or within 12 min (< 0.2 h) prior to the LSE protocol. While both groups had pre-test scores of 100% escape probability (n = 6 mice, 18/18 trials), the third group did not receive any prior test (Figures 3D and 3E).

We found that the group experiencing no pre-test exposure (who received the fewest number of overall looms) had the highest level of suppression following the LSE protocol (Figure 3F; n = 10 mice, escape on 1/30 [3.3%] trials). Regarding the pre-test groups, the group with a pre-test 24–26 h prior to the LSE protocol showed a small reduction of escape suppression (Figures 3E and 3F; no pre-test, 10 mice, 1/30 [3.3%)] trials versus 24–26 h pre-test, n = 6 mice, 4/18 [22.2%] trials, p = 0.06, Fisher’s exact test), while the 0.2 h pre-test group showed significantly higher escape probability (Figures 3E and 3F; no pre-test, n = 10 mice, 1/30 [3.3%] trials versus 0.2 h pre-test, n = 6 mice, 13/18 [72.2%] trials, p < 0.0001). Together, these data show that escape suppression induced by the LSE protocol is not simply explained by the number of stimulus exposures and that recent stimulus-escape experience modulates future escape behavior.

## Discussion

In the wild, prey animals often exhibit behavioral traits that do not appear to be learned, such as escaping from a threatening visual stimulus overhead. Here, we show that laboratory mice do not necessarily escape from their first or even subsequent exposures to looming stimuli but that robust escapes to shelter (from the same looming stimulus) can be reliably observed after only a 2-day period of individual housing. Such robust levels of escape behavior could therefore be consistent with potentially increased vigilance or heightened reactivity following social isolation.^22^^,^^23^ Other factors such as social density, hierarchy, and territoriality^24^^,^^25^ are also known to influence hormonal levels^26^ and behavior and may similarly impact escape behavior. We also show here that mice can quickly learn to suppress their otherwise robust escape and that this form of behavioral modification is persistent and dependent on recent stimulus-threat experience. Our data suggest that although the neuronal circuitry underlying escape at the age of weaning is hard wired, making the decision to escape is nevertheless a dynamic process that depends on both environmental^4^^,^^9^ and/or social factors and stimulus threat history. Such flexibility may be advantageous as a means to trade off competing behaviors such as vigilance versus resource acquisition (e.g., foraging, mating, or hunting).^27^^,^^28^ For example, as we also show here, mice will learn to modify their original defensive behavior elicited by novel prey insects to engage in prey capture.^6^

Recent studies have shown that the selection of a particular avoidance strategy can be flexible: for example, in scenarios where a shelter is or is not sufficiently close for successful escape.^4^ For instance, if a shelter is either absent or too distant, mice “choose” to freeze in response to predatory threat stimuli. In contrast, this study focuses on stimulus-experience-related modulation of escape decisions that operate on very different timescales. In the former scenario, decision-making is rapid and depends on information obtained from the local environment, and a decision is taken as to the optimal avoidance strategy (freeze or flee). Here, LSE operates over longer timescales (i.e., at least weeks) and reflects a re-evaluation of the level of threat and the decision of whether or not to ignore the stimulus.

In other species such as the pied flycatcher, the stimulus response (to dummy owls) can be seen to habituate at particular locations but can be readily elicited by the same stimulus in a new location.^29^ Our finding that escape responses of mice to novel live prey or artificial auditory stimuli presented in the same threat zone were unaltered following LSE implies that LSE under these experimental conditions does not reflect generalized learning about the safety of particular locations in the arena or the environment generally.^20^ Instead, LSE must relate to a stimulus feature or class of potential threat, and the memory of such properties is critically important for the selection of the behavioral response. LSE in mice may be more similar to Mongolian gerbils,^20^ which habituate their avoidance responses to particular visual stimuli and generalize across stimulus features rather than location.

Although we cannot say which LSE stimulus properties are critical for its induction, they may include contrast, number, interstimulus interval, and geometric shape. With this particular paradigm, the contrast ramp is not required for LSE (STAR Methods). Stimulus number is surely important, although the critical number of stimuli required remains to be determined. For example, we know that repetition of five high-contrast stimuli is not sufficient for robust LSE induction (data not shown). Regardless of what specific properties are important, LSE cannot be explained as a simple sensory desensitization mechanism. In fact, increasing the number of stimulus exposures by introducing a pre-test can prevent LSE. While the observed differences between species mentioned here may also be due in part to experimental designs, our data suggest that LSE is not simply habituation to repeated stimulus exposures but rather a distinct form of learning and memory of a specific set of stimulus features. These results have also revealed two ways in which recent experience, and therefore memory, impacts threat-avoidance-related decision-making. Firstly, LSE persists for at least 2 weeks, indicating long-term memory mechanisms ensure stable suppression of escape behavior. Secondly, we show that LSE itself can be occluded by the memory of either recent stimulus exposure or threat escape. Since LSE can also be induced using test-contrast stimuli (STAR Methods) where there is a partition preventing escape, it appears that it is the recent act of threat escape during the pre-test that prevents LSE. Our results also show that it is not simply the accumulation of stimulus exposures that triggers LSE induction. Perhaps the act of escape itself leads to a change in an internal state that prevents LSE whereby stimulus escape leads to short-term excitability or potentiation of the pathways that trigger escape for a particular sensory stimulus.

LSE likely arises due to changes in the neural circuitry mediating the decision to escape to a visual looming threat stimulus. The locus of escape initiation to such stimuli is thought to reside in the dorsal periaqueductal grey (PAG) and requires superior colliculus (SC) input to drive PAG neurons to threshold.^3^ Looming stimulus evoked activity in the medial SC scales with threat-stimulus salience,^3^ making it one possible candidate for LSE-based modulation. Indeed, a reduction of activity in SC has been shown to occur following repeated exposures of looming stimuli,^10^ although whether this is akin to LSE remains to be tested. Auditory escape, on the other hand, can be driven through cortico-collicular-PAG^11^ and direct cortico-lateral PAG pathways.^12^ Similarly, avoidance of novel prey may depend on a direct lateral hypothalamus to PAG pathway.^6^ One might predict that LSE leads to reduced activity in visual circuits of the SC without impacting on activity in the auditory pathways. If such a reduction in SC activity is involved in LSE, then structures upstream of the SC may be important. Visual areas of the cortex,^13^ thalamus,^14^ and the basal ganglia^30^ have all been shown to directly or indirectly modulate activity levels in the SC and could potentially regulate SC-mediated avoidance behaviors.

Behavioral adaptation requires the nervous system to update its predictive model of the world using ongoing sensory experience. We propose that LSE could provide a practical, laboratory-based yet ethologically relevant and robust model for modifying circuits that mediate flexible decision-making. Together with neurophysiological recordings and circuit dissection approaches,^3^^,^^6^^,^^10^^,^^15^^,^^31^, ^32^, ^33^ this could deliver an understanding of how the neural circuits underlying adaptive behaviors such as escape are under long-term cognitive control and modulated by experience to maximize fitness.

## STAR★Methods

### Key resources table

**Table:** 

REAGENT or RESOURCE	SOURCE	IDENTIFIER
**Deposited data**		

Preprocessed data	This paper	https://doi.org/10.5281/zenodo.6483652

**Experimental models: Organisms/strains**		

Mouse: C57BL/6J	Charles River	C57BL/6J
Black Crickets	Blades Biological	LZJ587

**Software and algorithms**		

lenzietal_lse_figures	This paper	https://github.com/SainsburyWellcomeCentre/lenzietal2022_lse_figures https://doi.org/10.5281/zenodo.6483625
looming_spots	This paper	https://github.com/SainsburyWellcomeCentre/looming_spots https://doi.org/10.5281/zenodo.6483482
DeepLabCut2.0	Mathis et al.^34^, Nath et al.^35^	https://doi.org/10.1038/s41593-018-0209-y https://doi.org/10.1038/s41596-019-0176-0
PsychToolBox	Brainard et al.^36^	N/A
MATLAB	The Mathworks	https://www.mathworks.com/
Python 3.6	Python Software Foundation	https://www.python.org

### Resource availability

#### Lead contact

Further information and requests for resources and reagents should be directed to and will be fulfilled by the lead contact, Troy W. Margrie (t.margrie@ucl.ac.uk).

#### Materials availability

• This study did not generate new unique reagents.

### Experimental model and subject details

#### Mice

Male C57BL/6J mice were obtained from commercial suppliers (Charles River) and were typically initially housed in cages of up to five littermates. For long-term group housing and enrichment experiments littermates were separated after weaning and placed either into an enlarged open pen (1 m x 1 m x 30 cm; groups of twenty, (GH, enriched)) or individually-housed in a standard IVC containing a Perspex shelter (IH IVC, 1 mth) for at least one month prior to testing. The pen contained running and climbing apparatus, multiple hiding spaces and shelters. For the individually-housed enriched condition (IH, enriched) after weaning mice were individually-housed in the same enlarged pen, with the same apparatus, for a period of one month. Finally, in the case of the short duration individually-housed condition, a single mouse aged between 6–14 weeks was removed from its litter and individually-housed in an IVC containing a shelter for between two to seven days. All food and water was provided ad lib. All procedures were performed in accordance with the UK Home Office regulations Animal (Scientific Procedures) Act 1986 and the Animal Welfare and Ethical Review Body (AWERB).

#### Crickets

Male black crickets were obtained from commercial suppliers (Blades Biological) and housed together in a terrarium. Each cricket was prepared at least twenty-four hours before use by attachment of a short (∼5–10 cm) piece of nylon cord with a small piece of magnetic tape at one end. Nylon cord was tied around each cricket’s thorax while the cricket was kept on ice. Crickets were then singly housed until the experiment. To ensure similar crickets were used in each condition, they were sorted into pairs of comparable size, and each of a pair allocated a different experimental group.

### Method details

#### Behavioural apparatus

The behavioural arena consisted of a red Perspex box (50 cm x 20 cm x 28 cm (L x W x H)) with a white opaque floor to provide high contrast to the dark coat of the mouse and ensure reliable tracking. At one end, the arena included either a dome (10 cm diameter) or a rectangular shelter (10 cm x 20 cm x 10 cm) made of red Perspex. An optional red Perspex partition could be inserted to isolate the “threat end” of the arena for the LSE protocol, 28 cm from the threat-end wall. IR LEDs provided diffuse illumination of the arena. Visual stimuli were presented using computer monitor (51 cm x 33 cm, Dell E2210F Black (WSXGA+) 22" TN) centred on the arena and positioned 30 cm above the floor, parallel with the floor to display visual stimuli.

#### Visual and auditory stimuli

Visual stimuli were generated using PsychToolBox^36^ and MATLAB (The Mathworks). A single test looming stimulus consisted of five high contrast expanding spots (Figure 1A), which each expanded linearly from 3 to 50 degrees over 0.2 s (220 deg/s) and remained at maximum radius for 0.25 s. The inter-spot-interval was 0.4 s and the inter-stimulus-interval was 90 s. The Weber contrast of looming spots (luminance: 0.13 cd/m^2^) used for testing escape was −0.98.

For the LSE protocol and beginning with the lowest contrast, stimuli were presented in a set of three with an inter-stimulus-interval of forty seconds and repeated for eight different increasing contrast levels (Figure 2A).

Auditory threat stimuli consisted of 85 dB pink noise for three seconds presented over the sound card (Realtek) using MATLAB, an amplifier (qtx-kad2), and a loudspeaker (8 Ohm, 10 W) attached to the far end of the monitor above the threat zone facing downwards.

#### Behavioural procedures

All mice were tested during the dark phase of the housing light cycle. Individual mice were either transferred in their home IVC or removed from the enlarged pen and placed in an IVC before being transported to the experimental room and given at least five minutes to acclimatize under low light conditions. After this time, mice were transferred to the behavioural arena the computer monitor positioned overhead. Either a curtained enclosure or an anechoic chamber (120 cm (L) x 90 cm (W) x 100 cm (H), LS Fabrications) was then closed and unless otherwise stated, the mouse was given seven minutes to explore the entire arena and shelter.

#### LSE protocol

For LSE experiments mice were introduced to the threat zone of the behavioural arena that was partitioned using an opaque Perspex wall (28 cm from the threat-end wall) preventing the mouse from seeing or reaching the shelter. Once in the behavioural arena, the stimulus monitor was positioned overhead and the LSE protocol was immediately initiated. After the LSE protocol, the partition was removed, and mice were allowed 7 minutes to explore the entire arena and shelter before being probed with looming stimuli at testing contrast. In a subset of experiments testing the longitudinal effects of LSE, mice were immediately removed from the arena after the LSE protocol and returned to their home cage to be tested at a later date. Robust LSE was also observed when using the same number of stimuli all at test-stimulus contrasts (post-LSE escape, n = 8 mice, 1/24 (4.2%) trials).

#### Live prey experiments

Mice were introduced to the restricted threat zone arena and were either presented with the LSE protocol or a constant grey screen (luminance: 7–8 cd/m^2^) for an equivalent period of time (16 mins). After this, the partition was removed, and mice were given seven minutes to explore the full arena before a cricket was introduced and tethered by the magnetic tape to a magnet placed at the centre of the threat zone. Mice were then given four hours to interact with the cricket.

#### Data acquisition

Data acquisition was controlled using custom scripts in MATLAB or Python and a NIDAQ (National Instruments, BNC2090A and PCI-6363). Videos were acquired at 30 (escape and LSE), 50 (cricket) or 100 (mouse orientation) frames per second using an IR sensitive camera (Basler acA640-750 um USB 3.0) positioned 70 cm away from the arena and 70 cm above the floor of the arena. Frame acquisition was triggered using a NIDAQ generated TTL that was also recorded and used for post-hoc synchronisation. Online tracking of the mouse centre of mass was used to trigger the presentation of stimuli when mice entered a “threat zone”, a 20 cm x 20 cm region at the opposite end of the arena to the shelter. Some data were acquired by manually triggering the stimulus upon entry to the threat zone. Real-time stimulus presentation onsets were determined post-hoc using a photodiode (Thorlabs APD430C) and the TTL trigger acquired at 10 kHz.

#### Behavioural tracking

Videos were tracked offline in two dimensions using DeepLabCut2.0.^34^^,^^35^ Three different networks were used for tracking – one for escape and LSE experiments with a single label for tracking the centre of mass of the mouse, a second used additional labels (shoulder blades, left ear and right ear) to estimate the mouse heading angle and a third for tracking both the mouse and cricket positions. All networks were trained using the default network (Resnet_v1_50) and training was always run to completion (> 1000000 iterations with a final loss of 0.001). Tracks were filtered using DeepLabCut’s built-in median filtering with a window length of 5 frames. Sixty-four videos were suboptimal for tracking and were tracked manually.

#### Classification of behavioural responses

Positional tracks were perspective-corrected using a projective transformation applied using the coordinates of the corners of the arena and its known geometry and were converted to cm from pixels using the known dimensions of the arena.

Analytical metrics were derived from the Gaussian smoothed positional trace of the mouse along the longest edge of the arena (x-position). Speed was calculated as the smoothed differential of this positional track. Acceleration was the double differential of this positional track, and latency to escape was defined as the time from stimulus onset to the onset of the first trajectory back to shelter. Peak speed was defined as the maximum speed reached towards shelter following stimulus onset. Time-to-shelter was defined as the time from stimulus onset to the mouse reaching the front threshold of the shelter.

Escape responses were classified as a return to shelter within five seconds of stimulus onset exceeding a speed of 25.5 cm/s towards shelter at any point within five seconds of stimulus onset. These criteria were derived from all mice tested on the set up, using trials considered to be escapes by an experimenter and taking the average time to shelter of the 5 latest returns to shelter and the average peak speed of the 5 slowest trials respectively. Freezing was defined as a difference of less than 2.5 cm/s between the 2.5^th^ and 97.5^th^ percentiles of all speed values measured from 0.4 s until five seconds after stimulus onset and was determined using all freeze responses observed and that were first manually classified. Traces that were not classified as escape or freezing were classified as no-reactions.

The escape robustness for a mouse was defined as:

$$Escape\;robustness=\frac{escape\;probability*avg.\;escape\;speed}{avg.\;latency\;to\;shelter}$$

Heading angle was derived from the 2D tracked positions of the left ear $\left(P_{leftear}\right)$, the right ear $\left(P_{rightear}\right)$ and the shoulders $\left(P_{shoulders}\right)$ using the following formula:

$$angle\;from\;shelter=\left(\frac{180}{\pi }\right)\cos^{-1}\left(\frac{\bar{H}.\bar{u}}{\left|H\right|\left|u\right|}\right)$$

Where $\bar{H}$ is the vector pointing from the shoulders through the middle of the ears and is defined as:

$$\bar{H}=\frac{P_{left\;ear}+P_{right\;ear}}{2}-P_{shoulders}$$

And $\bar{u}$ is the vector pointing along the x-axis toward the shelter and away from the “threat-end” of the arena, defined as:

$$\bar{u}=\left(\begin{array}{l} -1\\ 0 \end{array}\right)$$

Resulting in an angle between 0 and 180 degrees where 0 points along the x-axis directly towards shelter and 180 directly away from it.

To compute stimulus evoked changes in the heading angle (Figure 2C) of naïve mice, the average baseline heading angle (relative to the shelter location) was determined for the 200 ms immediately before stimulus onset. The minimum angle was then determined for the period of time from stimulus onset until arrival at the shelter. The baseline angle was then subtracted from the post-stimulus angle. In the two cases where the mouse did not return to shelter (one freeze and one no-reaction), the longest post-stimulus period measured in escape trials was used. Since LSE mice did not show escape, post-stimulus analysis windows for this group were pseudorandomly matched in duration with those obtained from the naïve group.

For cricket experiments, interactions between the mouse and cricket were defined as the Euclidean distance between the mouse and cricket body positions being less 10 cm. A bout was defined as the two seconds following an interaction and classified as a return to shelter if the mouse reached the front edge of the shelter within this time window.

### Quantification and statistical analysis

Analysis was performed using custom python code. Statistical details of all experiments can be found in the main text of the Results section. Stats were computed in Python using the SciPy stats module or the Circular Statistics Toolbox in MATLAB.^26^ For all tests, results were considered significant if p < 0.05. The following statistical tests were used: Fisher’s exact test, Kuiper’s test, Wilcoxon’s rank-sum test, and Chi-squared test.

## Data Availability

• Processed data have been deposited at Zenodo and are publicly available as of the date of publication. DOIs are listed in the key resources table. • All original code has been deposited at Zenodo and is publicly available as of the date of publication. DOIs are listed in the key resources table. • Any additional information required to reanalyse the data reported in this paper is available from the lead contact upon request.

## Supplemental information

Document S1. Figures S1 and S2

Document S2. Article plus supplemental information
