# Supplementary material for: Threat history controls flexible escape behavior in mice
Source: Curr Biol. 2022 Jul 11;32(13):2972–2979.e3. doi: 10.1016/j.cub.2022.05.022 (PMC9616793; doi:10.1016/j.cub.2022.05.022)
Supplement: Document S1. Figures S1 and S2 [file mmc1.pdf]

Current Biology, Volume *32*

## Supplemental Information

# Threat history controls flexible escape behavior in mice

**Stephen C. Lenzi, Lee Cossell, Benjamin Grainger, Sarah F. Olesen, Tiago Branco, and Troy W. Margrie**

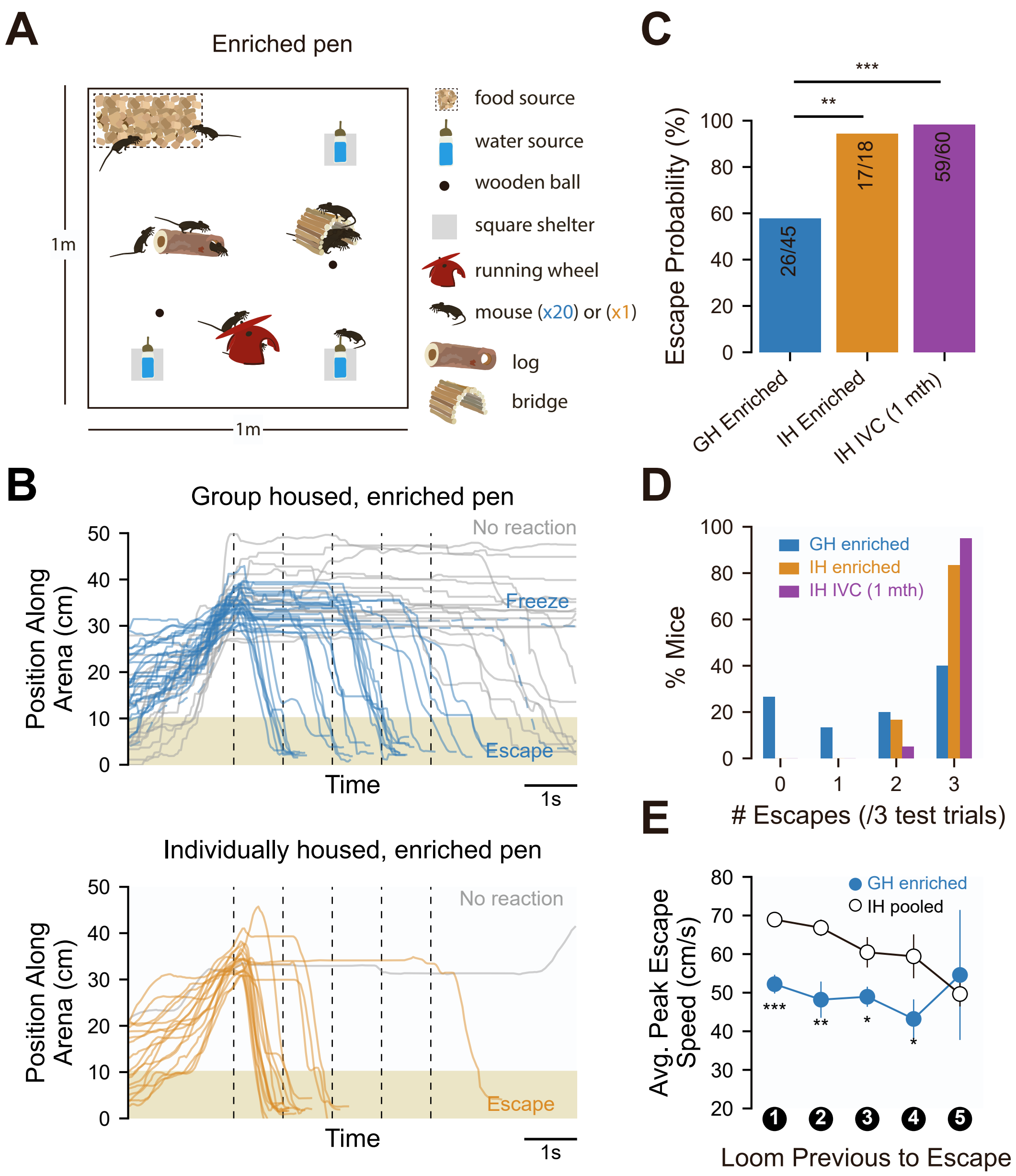

A
Enriched pen
1m
1m
food source
water source
wooden ball
square shelter
running wheel
mouse (x20) or (x1)
log
bridge
B
Group housed, enriched pen
No reaction
Freeze
Escape
Position Along Arena (cm)
Time
1s
Individually housed, enriched pen
No reaction
Escape
Position Along Arena (cm)
Time
1s
C
Escape Probability (%)
26/45
17/18
59/60
**
***
GH Enriched
IH Enriched
IH IVC (1 mth)
D
% Mice
GH enriched
IH enriched
IH IVC (1 mth)
# Escapes (/3 test trials)
E
Avg. Peak Escape Speed (cm/s)
GH enriched
IH pooled
***
**
*
*
Loom Previous to Escape

**Figure S1: Unreliable escape behaviour in group housed mice. Related to Figure 1. A**) Schematic illustrating the large environmentally enriched pen in which mice were either individually housed or housed as a group (n = 20) for at least 28 days before testing. **B**) Top: population data showing positional traces of all trials for all group housed mice (traces are classified as escape (solid line), freezing (dashed line) or no reaction (solid grey line). Bottom: population data showing positional traces of all trials for all individually housed mice. **C**) Overall percentage of trials classified as escape according to housing condition. ** and *** indicate p-values of less than 0.01 and 0.001 respectively. **D**) Percentage of mice which escaped to 0, 1, 2 or 3 of the three test trials, shown for each experimental group. **E)** The mean peak speed for all escape trials grouped by the most recent looming spot preceding escape initiation for group housed and isolated mice (pooled with individually housed IVC conditions). *, ** and *** indicate p-values of less than 0.05, 0.01 and 0.001 respectively. Error bars indicate standard error of the mean (SEM).

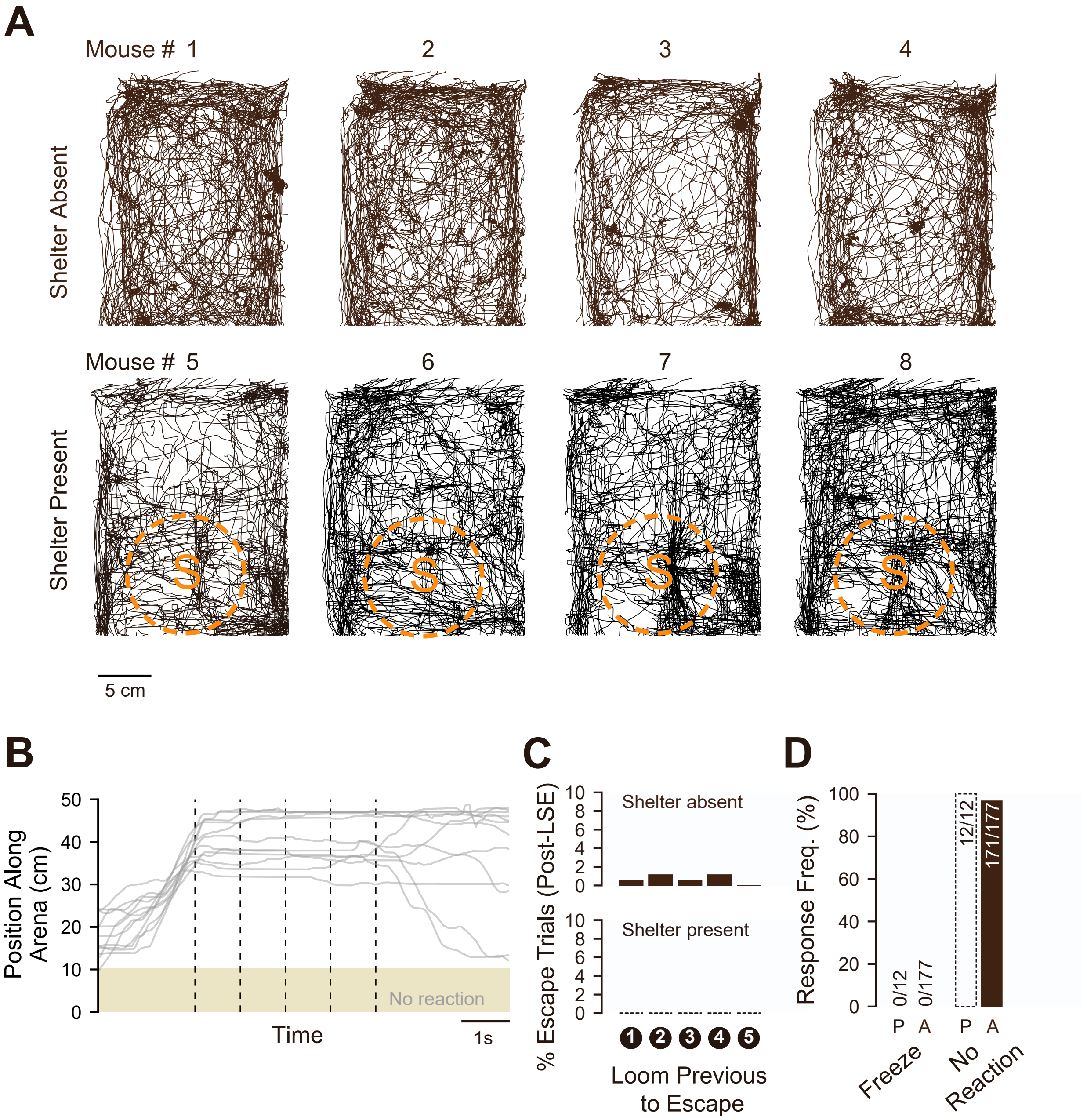

A
Mouse # 1
2
3
4
Shelter Absent
Mouse # 5
6
7
8
Shelter Present
S
5 cm
B
Position Along Arena (cm)
50
40
30
20
10
0
No reaction
Time
1s
C
% Escape Trials (Post-LSE)
Shelter absent
Shelter present
10
8
6
4
2
0
1
2
3
4
5
Loom Previous to Escape
D
Response Freq. (%)
100
80
60
40
20
0
0/12
0/177
12/12
171/177
P
A
P
A
Freeze
No Reaction

**Figure S2: Access to shelter during the LSE protocol does not influence its induction. Related to Figure 2. A)** Trajectory plots for four example mice during the LSE protocol when a shelter was absent (brown, top), or present (black, bottom). **B)** Positional traces for all trials from the four mice shown in **A**) (bottom) recorded during the post-LSE test trials. **C)** Bar graphs showing the percentage of trials classified as escape, grouped by most recent looming spot preceding escape initiation for each experimental group. **D)** The percentage of responses classified as freezing or as no-reaction for each experimental group.
